# Use of the margin of stability to quantify stability in pathologic gait – a qualitative systematic review

**DOI:** 10.1186/s12891-021-04466-4

**Published:** 2021-06-28

**Authors:** Fraje Watson, Peter C. Fino, Matthew Thornton, Constantinos Heracleous, Rui Loureiro, Julian J. H. Leong

**Affiliations:** 1 University College London, Division of Surgery & Interventional Science, Royal National Orthopaedic Hospital, Brockley Hill, Stanmore, HA7 4LP UK; 2 Department of Health & Kinesiology, University of Utah, 250 S 1850 E, Salt Lake City, UT 84112 USA; 3 Royal National Orthopaedic Hospital, Brockley Hill, Stanmore, HA7 4LP UK

**Keywords:** Margin of stability, Dynamic stability margin, Extrapolated Centre of Mass, XcoM, Base of support, Stroke, Transtibial amputation

## Abstract

**Background:**

The Margin of Stability (MoS) is a widely used objective measure of dynamic stability during gait. Increasingly, researchers are using the MoS to assess the stability of pathological populations to gauge their stability capabilities and coping strategies, or as an objective marker of outcome, response to treatment or disease progression. The objectives are; to describe the types of pathological gait that are assessed using the MoS, to examine the methods used to assess MoS and to examine the way the MoS data is presented and interpreted.

**Methods:**

A systematic review was conducted in accordance with the Preferred Reporting Items for Systematic Reviews and Meta-Analyses Guidelines (PRISMA) in the following databases: Web of Science, PubMed, UCL Library Explore, Cochrane Library, Scopus. All articles measured the MoS of a pathologically affected adult human population whilst walking in a straight line. Extracted data were collected per a prospectively defined list, which included: population type, method of data analysis and model building, walking tasks undertaken, and interpretation of the MoS.

**Results:**

Thirty-one studies were included in the final review. More than 15 different clinical populations were studied, most commonly post-stroke and unilateral transtibial amputee populations. Most participants were assessed in a gait laboratory using motion capture technology, whilst 2 studies used instrumented shoes. A variety of centre of mass, base of support and MoS definitions and calculations were described.

**Conclusions:**

This is the first systematic review to assess use of the MoS and the first to consider its clinical application. Findings suggest the MoS has potential to be a helpful, objective measurement in a variety of clinically affected populations. Unfortunately, the methodology and interpretation varies, which hinders subsequent study comparisons. A lack of baseline results from large studies mean direct comparison between studies is difficult and strong conclusions are hard to make. Further work from the biomechanics community to develop reporting guidelines for MoS calculation methodology and a commitment to larger baseline studies for each pathology is welcomed.

## Background

Stable gait is important in order to maintain active living, and various methods to measure gait stability are reported throughout the literature [1]. Many neuromuscular conditions and physical abnormalities (e.g., amputations) can impair the ability to regulate balance and subsequently impair independence [2, 3]. Effectively quantifying stability in these clinical populations has gained significant interest as increased knowledge of balance deficits or compensatory strategies may aid rehabilitation and inform strategies to mitigate associated risks such as falling.

**Fig. 1:**
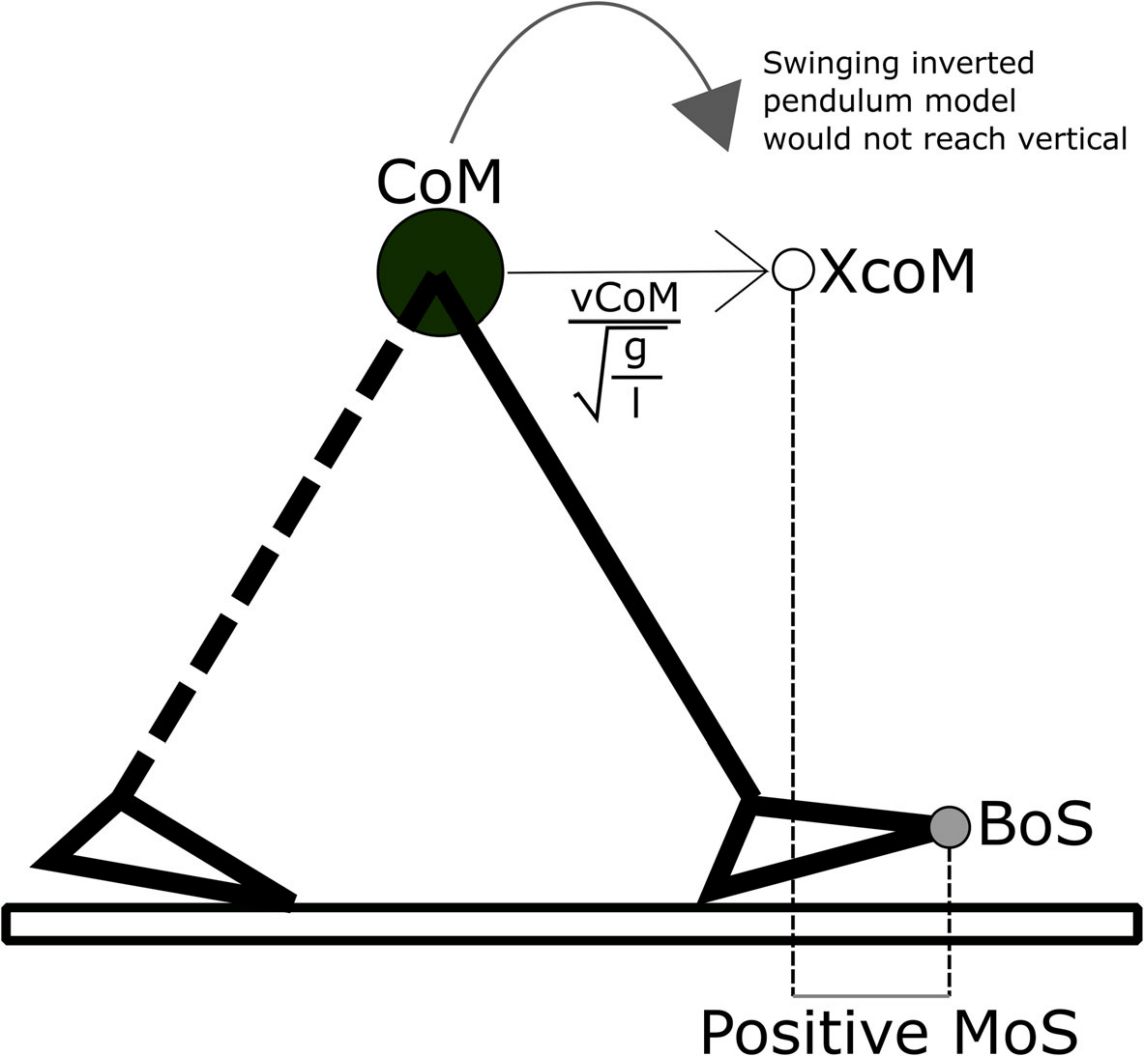
An inverted pendulum model shown at the point of heel strike An inverted pendulum model shown at the point of heel strike can be used to consider the anterior-posterior (AP) Margin of Stability (MoS). The inverted pendulum consists of a mass (black circle) representing the Centre of Mass (CoM) atop a very light rod representing the leg length (solid black) attached to a hinge at the ground representing the ankle joint. The Extrapolated CoM (XcoM) (white circle) is the position of the CoM accounting for velocity, as described in more detail in text. The anterior limit of the Base of Support (grey circle) is positioned at the toe. In this example the XcoM is behind the BoS and calculating the MoS_AP_ (BoS - XcoM) would give a positive value. Under these conditions and given the CoM is the origin, the Inverted Pendulum model would fail to reach vertical and would eventually return to it’s current position, which is considered “stable” for this systematic review. Conversely, if the XcoM was in front of the BoS the inverted pendulum would swing over vertical and proceed to fall to the ground (rightward on the planar image) – it would not return to its current position and is therefore considered unstable for this systematic review. This figure was created by FW

Balance control during walking is accomplished by constantly regulating the location of the body’s centre of mass (CoM) with respect to the area encompassed by the feet (base of support [BoS]). In bipeds, the CoM is set high over a small BoS, meaning that even small body position changes can have great effect on the motion of the CoM, requiring expert control [4]. Winter (1995) [5] described stable gait in anterior-posterior (AP) and mediolateral (ML) directions during standing and walking using an inverted pendulum model. In the inverted pendulum model, a mass (e.g., the body CoM) is positioned atop a light, rigid rod (e.g., a leg) and secured to the ground at a hinge (e.g., the ankle) on which it oscillates back and forth. At that time it was accepted that stability could be maintained by positioning the CoM within the BoS [5], but Pai, et al. (1997) [6] identified that this theory was not conducive to dynamic situations. In response, Hof, et al. (2005) [7] introduced the extrapolated CoM (XcoM). The XcoM is an estimation of the CoM projected on the ground, combined with its velocity, and standardized by the pendulum length (e.g. height of the CoM),

Equation 1: XcoM calculation

$$ XcoM= CoM+\frac{vCoM}{\sqrt{\frac{g}{l}}} $$

where vCoM is the velocity of the CoM, *g* is the gravitational acceleration and *l* is the height of the pendulum. In 2008, Hof [8] proposed that control of the XcoM position with respect to the BoS (defined as the possible range of the centre of pressure [CoP]) was vital for walking stability. Subsequently, the term Margin of Stability (MoS) was coined to quantify the relationship between the XcoM and the BoS,

Equation 2: MoS calculation

$$ MoS= BoS- XcoM $$

where the *BoS* and *XcoM* are position vectors with origins at the position of the CoM. By incorporating the XcoM into the inverted pendulum model (Fig. 1) we can describe and predict stability, i.e. the systems instantaneous mechanical stability [9]. When the MoS is positive, the pendulum will not rotate over vertical, and will instead return back to its current position, which we consider to reflect a positive stability. Such a scenario is depicted in Fig. 1. At the point of gait shown (heel strike), the XcoM is positioned within the BoS and the MoS in the AP direction, MoS_AP_ will be positive and considered stable because the pendulum would not proceed beyond vertical if no further forces other than that of gravity are applied. Conversely, if the XcoM was positioned beyond the BoS, the MoS_AP_ would be negative and considered unstable because the pendulum would continue to swing beyond vertical and would not return to its original position. Thus, when the CoM is closer to the XcoM than to the BoS, we can define a positive MoS as stable (i.e., the body as a pendulum would return to its current position without intervention). As discussed later, an important consideration is the *direction of instability*. For a backwards loss of balance and in a standard reference frame with anterior displacement being positive, the MoS calculation would yield a *negative* value when in a stable configuration (i.e., the position of the BoS would be more negative than the position of the XcoM). Thus, some authors flip the order of subtraction (e.g., XcoM – BoS) to preserve the positive = stable relationship. However, this calculation can lead to confusion in interpretation between papers, despite an engaging case for the preference of either. Due to the absence of biomechanical consensus with regards to the MoS using the inverse pendulum model, the MoS will be calculated and interpreted per Eq. 2 in this paper.

Since 2008, the MoS, sometimes termed the Dynamic Stability Margin among other similar terms, has been increasingly used by researchers in healthy and pathologic [10–14] populations, during straight line walking [15], turning [16], rehabilitation [17] and for perturbation response [18]. The MoS is most commonly measured using a kinematic gait laboratory, but options for measurement with wearable devices are emerging [9, 19, 20]. Throughout these studies, the calculations that contribute to the MoS have been interpreted differently or not explicitly described across the literature, making direct comparisons and interpretations between papers studying the same clinical population difficult for clinicians and researchers alike.

The objectives of this systematic review were to describe the types of pathological gait that have been assessed using the MoS, to examine the methods used to assess MoS and to comment on data interpretation and results.

## Methods

### Protocol and registration

The protocol for this review was registered at University College London’s research data repository (10.5522/04/12102900.v1).

### Eligibility criteria

Studies were eligible if they were published between 2005 and 2020. The start date was chosen because it was the year of publication of a seminal paper [7] in the field, which contributed towards the existence of the MoS as it is known today. The Preferred Reporting Items for Systematic Reviews and Meta-Analyses (PRISMA) [21] were used.

Included studies were required to be written in English or fully translated. Included studies were those that assessed the MoS in an adult, human population with a pathological condition, e.g. with Parkinson’s or a trans-tibial amputee. Pregnancy, obesity, and age were not considered pathological afflictions, except for papers including an elderly faller population. Included studies measured MoS during straight-line walking. Studies that analysed specific gait events or types (e.g. gait initiation, gait termination, turning) or that assessed the impact of training or rehabilitation on the MoS were included if the paper also included and described data for a straight-line walk (e.g. as a baseline).

### Information sources

Five databases were searched; Web of Science, PubMed, UCL Library Explore, Cochrane Library and Scopus. Key words included the following search terms: (a) dynamic stability margin, dynamic gait stability, margins of stability or margin of stability, (b) center of mass, centre of mass, center of pressure, centre of pressure, and (c) base of support, which were combined into (a) AND (b) AND (c). “All fields” were specified and sources years between January 2005 and March 2020 were selected. Theses were excluded, but a separate search for resulting publications was performed and included if they met the criteria. Books, newspaper articles and review articles were excluded. Finally, references of included articles were searched to ensure that the electronic records had not overlooked relevant articles. Authors of included articles were not contacted for additional information or to identify additional studies for inclusion. A methodologist or specialist librarian was not consulted to help this search.

### Search

As an example, Scopus was searched using the following query:

(((ALL (“dynamic stability margin”) OR ALL (“dynamic stability”) OR ALL (“dynamic gait stability”) OR ALL (“dynamic balance control”)) AND PUBYEAR > 2004) OR ((ALL (“margin of stability”) OR ALL (“margins of stability”)) AND PUBYEAR > 2004)) AND ((ALL (“center of mass”) OR ALL (“centre of mass”) OR ALL (“center of pressure”) OR ALL (“centre of pressure”)) AND PUBYEAR > 2004) AND (ALL (“base of support”) AND PUBYEAR > 2004)

### Study selection

One reviewer (FW) conducted a systematic search for publications between January 2005 and March 2020. Duplicates were removed and, when appropriate, journal papers were selected over conference papers. Once duplicates were removed, two reviewers (FW & CH) assessed each reference based on title, abstract or full text, as necessary to ensure adherence with the inclusion/exclusion criteria. Where reviewers could not agree on the inclusion/exclusion of certain papers, a third reviewer (JL) made the decision.

### Data collection process and items

Data was collected by a single reviewer (FW) using a pre-defined checklist which included: clinical population, number of affected participants, age, weight, sex and height of affected participants, inclusion of a control group, equipment used to measure MoS, marker number, walking speed, walking task specifics, method for defining the CoM, pendulum height definition, definition of the BoS, definition of the MoS and at what point that measurement was extracted, and brief results pertaining to the MoS during straight-line walking.

### Risk of bias in individual studies and across studies

A National Institutes of Health quality assessment tool for a case-control and cohort/cross-sectional study [22] was used to assess risk of bias. As seen in Table 1, eleven studies were rated “good”, twelve studies were rated as “fair”, and eight papers were rated as “poor”. The most common elements that introduced risk were failure to justify a sample size, failure to describe the recruitment of participants (particularly place and time period), failure to describe the inclusion/exclusion criteria for the control group and failure to describe how many participants were eligible for recruitment or approached for recruitment or how participants were selected at all. Study objectives, pathologic participants and outcome measures were generally well described. In terms of risk of bias across studies, the included studies all involve an affected clinical population and, therefore, it is possible that MoS methodology and reporting of results was adapted to best suit a specific population’s characteristics and equipment available at the establishment.

**Table 1:** Risk of bias in individual studies quality assessment

Paper	Case-Control Criteria										Cohort or Cross-Sectional Criteria								Grade
	1^**1**^	2^**2**^	3^**3**^	4^**4**^	5^**5**^	6^**6**^	7^**7**^	8^**8**^	9^**9**^	10^**10**^	1^1^	2^2^	3^**11**^	4^**12**^	5^3^	6^**13**^	7^9^	8^10^	
Hof, et al. (2007) [34]	Y	N	N	NR	NR	N	CD	CD	N	NR									Poor
Curtze, et al. (2011) [33]	Y	N	N	NR	NR	N	CD	CD	N	N									Poor
Day, et al. (2012) [38]	Y	Y	N	N	Y	Y	NR	N	N	Y									Good
Stegemöller, et al. (2012) [37]	Y	N	N	NR	Y	Y	CD	CD	N	Y									Good
Gates, et al. (2013) [32]	Y	N	N	NR	NR	N	CD	CD	N	Y									Fair
Hak, et al. (2013) [31]	Y	Y	N	N	NR	N	CD	CD	N	Y									Fair
Hak, et al. (2013) [14]	Y	Y	N	N	NR	N	CD	CD	N	Y									Fair
Major, et al. (2013) [3]	Y	Y	N	NR	NR	N	CD	CD	N	CD									Fair
Beltran, et al. (2014) [30]	Y	N	N	NR	NR	N	CD	CD	N	N									Poor
Hak, et al. (2014) [28]											Y	N	NR	NR	N	Y	N	N	Fair
Kao, et al. (2014) [27]	Y	N	N	NR	NR	N	CD	CD	N	Y									Fair
McCrum, et al. (2014) [18]	Y	N	N	NR	Y	Y	CD	CD	N	CD									Good
Hak, et al. (2015) [23]											Y	Y	NR	NR	N	Y	N	Y	Good
Hoogkamer, et al. (2015) [44]	Y	N	N	NR	NR	N	CD	CD	N	N									Poor
Rijken, et al. (2015) [40]	Y	Y	N	N	N	N	CD	CD	N	CD									Fair
Catalá, et al. (2016) [36]	Y	N	N	NR	NR	N	CD	CD	N	CD									Poor
Peebles, et al. (2016) [12]	Y	N	N	NR	Y	Y	CD	CD	N	N									Fair
van Meulen, et al. (2016) [20]											Y	Y	NR	NR	N	Y	N	Y	Good
van Meulen, et al. (2016) [19]											Y	Y	NR	NR	N	Y	N	Y	Good
Vistamehr, et al. (2016) [26]											Y	N	NR	NR	N	Y	N	CD	Fair
Ghomian, et al. (2017) [43]											Y	n/a	n/a	n/a	N	Y	N	n/a	Poor
Martelli, et al. (2017) [11]	Y	N	N	CD	N	N	CD	CD	N	CD									Poor
Peebles, et al. (2017) [39]	Y	N	N	CD	Y	Y	CD	CD	N	Y									Good
Punt, et al. (2017) [24]											Y	Y	NR	NR	N	Y	N	Y	Good
Simon, et al. (2017) [42]	Y	Y	N	CD	Y	Y	CD	CD	N	CD									Good
Tisserand, et al. (2018) [25]	Y	Y	N	N	N	Y	CD	CD	N	N									Fair
Arora, et al. (2019) [13]	Y	Y	Y	N	N	Y	CD	CD	N	N									Good
Brandt, et al. (2019) [29]											Y	Y	NR	NR	N	Y	N	N	Fair
Major, et al. (2019) [35]											Y	N	NR	NR	N	Y	N	N	Poor
van Vugt, et al. (2019) [41]	Y	Y	N	N	N	N	CD	CD	N	N									Fair
de Jong, et al. (2020) [45]	Y	Y	N	Y	Y	Y	CD	CD	N	N									Good

Y = Yes; N = No; NR = Not Reported; CD = Cannot Determine; n/a = Not Applicable

^1^Clear and appropriate research question?

^2^Study population clearly defined?

^3^Sample size justification?

^4^Controls selected from same population as cases?

^5^Inclusion/exclusion criteria clear, reliable, consistent?

^6^Cases clearly differentiated from controls?

^7^Randomly selected from eligible participants?

^8^Concurrent controls?

^9^Assessors blinded?

^10^Confounding variables measured and accounted for in statistics?

^11^Participation of eligible participants at least 50%?

^12^Subjects selected from same population?

^13^Outcome measures clearly defined, reliable, valid?

## Results

### Study selection

**Fig. 2:**
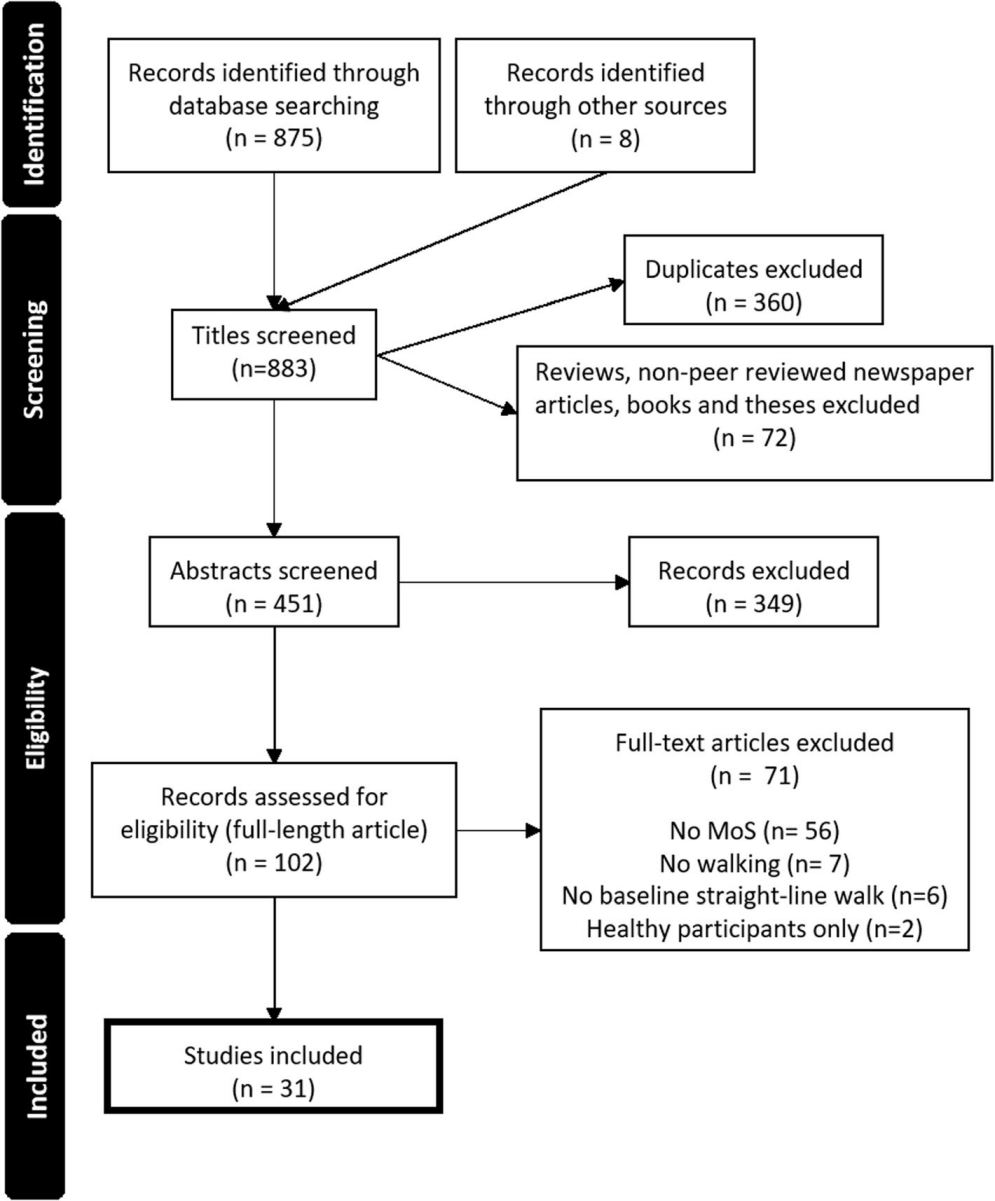
PRISMA flowchart detailing the literature search and study selection

In total 883 records were identified: 875 from aforementioned databases and 8 from theses and reference lists of included articles (Fig. 2). This list contained 360 duplicate articles and 72 reviews, non-peer-reviewed articles, books, and theses, which left 451 articles for screening. Three-hundred forty-nine records were excluded based on the abstract alone mostly because they only included healthy participants, leaving 102 full-length articles for consideration. Seventy-one full-length articles did not meet the inclusion criteria because, either: they did not use the MoS (*n* = 56), participants did not walk (*n* = 7), they considered other aspects of walking (e.g., rehabilitation training, turning) and did not include a baseline straight walk (*n* = 6), or included only healthy participants (*n* = 2). Thirty-one articles were included in this systematic review.

### Study characteristics

Table 2 lists the cohort pathology, cohort size, presence of a control group, age, height and weight of affected cohort and details how controls were matched. Table 3 lists the equipment used to measure the MoS, the walking tasks performed and the gait speed. Table 4 lists variables pertaining to XcoM and MoS calculation, original author results and a standardised interpretation of results to reflect the definition of stability given in the introduction.

**Table 2:** Summary of pathologies, affected cohort and use of controls

Paper	Cohort pathology	Affected cohort size	Affected cohort sex (F:M)	Affected cohort age (years) [Mean (SD)]	Affected cohort mass (Kg) [Mean (SD)]	Affected cohort height (m) [Mean (SD)]	Control group?	Control group matching
**Stroke (*n*** **= 8)**								
Hak, et al. (2013) [14]	Stroke Left hemiparesis (n = 6) Right hemiparesis (*n* = 4) Acute (n = 4) Chronic^14^ (n = 6)	10	Not specified	60.8 (8.4)	88.4 (8.5)	1.79 (0.07)	Yes	Age
Kao, et al. (2014) [27]	Stroke Chronic (*n* = 9)	9	4:5	60.8 (9.0)	Not specified	Not specified	Yes	Age, sex
Hak, et al. (2015) [23]	Stroke Left hemiparesis (n = 5) Right hemiparesis (*n* = 5) Acute (*n* = 6) Chronic (*n* = 4)	10	4:6	57.6 (15.4)	77.9 (16.5)	1.72 (0.11)	No	n/a
van Meulen, et al. (2016) [20]	Stroke Left hemiparesis (*n* = 10) Chronic (*n* = 10)	10	3:7	63.2 (8.9)	91.0 (9.8)	1.74 (0.09)	No	n/a
van Meulen, et al. (2016) [19]	Stroke Left hemiparesis (*n* = 11) Right hemiparesis (*n* = 2) Chronic (*n* = 13)	13	5:8	64.1 (8.7)	87.67 (10.47)	1.73 (0.10)	No	n/a
Vistamehr, et al. (2016) [26]	Stroke Left hemiparesis (*n* = 16) Right hemiparesis (*n* = 3) Chronic (*n* = 19)	19	6:13	62.0 (11.0)	Not specified	Not specified	No	n/a
Punt, et al. (2017) [24]	Stroke Left hemiparesis (*n* = 12) Right hemiparesis (*n* = 26) Chronic (*n* = 38)	38	20:18	Non-fallers: 55.0 (12.2) Fallers: 65.4 (6.7)	Non-fallers: 87.0 (19.0) Fallers: 83.0 (20.1)	Non-fallers: 1.72 (0.10) Fallers: 1.71 (0.13)	No	n/a
Tisserand, et al. (2018) [25]	Stroke Left hemiparesis (*n* = 4) Right hemiparesis (n = 8) Chronic (*n* = 10)	12	5:7	58.2 (10.0)	85.5 (35.5)	1.66 (0.17)	Yes	Age, anthropometric parameters
**Unilateral Transtibial Amputation (n = 5)**								
Curtze, et al. (2011) [33]	Unilateral transtibial amputation Traumatic (n = 11) Vascular disease (n = 6) Limb deficiency (n = 1)	18	0:18	55.6 (9.5)	90.3 (14.37)	1.83 (0.05)	Yes	Not specified
Gates, et al. (2013) [32]	Unilateral transtibial amputation Traumatic (n = 13)	13	1:12	28.0 (4.0)	88.6 (14.4)	1.81 (0.09)	Yes	Not specified
Hak, et al. (2013) [31]	Unilateral transtibial amputees Traumatic (*n* = 9) Complex regional pain syndrome (*n* = 1)	10	1:9	38.8 (14.6)	87.1 (10.3)	1.83 (0.11)	Yes	Age
Beltran, et al. (2014) [30]	Unilateral transtibial amputees Traumatic (n = 9)	9	0:9	30.7 (6.8)	90.2 (16.1)	1.76 (0.11)	Yes	Not specified
Hak, et al. (2014) [28]	Unilateral transtibial amputees Traumatic (*n* = 8), Dysvascular (n = 1) Other (*n* = 1)	10	1:9	38.8 (14.6)	87.1 (9.76)	1.83 (0.11)	No	n/a
**Other amputation (n = 4)**								
Hof, et al. (2007) [34]	Unilateral transfemoral amputation	6	2:4	40.5 (6.0)	69.3 (19.1)	1.74 (0.08)	Yes	Leg length, mass, sex
Major, et al. (2013) [3]	Bilateral transtibial amputees Vascular (*n* = 5) Traumatic (n = 3) Congenital (*n* = 1) Meningitis (n = 1)	10	Not specified	50.0 (18.0)	82.0 (16.0)	1.73 (0.08)	Yes	Age, gait speed
Brandt, et al. (2019) [29]	Unilateral transfemoral amputee or knee disarticulation Trauma (n = 3) Cancer (n = 2) Congenital (n = 1)	6	1:5	40.8 (19.7)	68.2 (13.5)	1.75 (0.05)	No	n/a
Major, et al. (2019) [35]	Transradial and transhumeral amputees Transradial (n = 7) Transhumeral (n = 3)	10	3:7	50.0 (19.0)	75.3 (18.6)	1.75 (0.08)	No	n/a
**Spinal cord injury (SCI) (n = 2)**								
Day, et al. (2012) [38]	SCI Cervical (n = 7) Thoracic (n = 3)	10	4:6	42.6 (14.2)	Not specified	Not specified	Yes	Not specified
Arora, et al. (2019) [13]	SCI Tetraplegic (n = 11) Paraplegic (n = 9)	20	5:15	60.1 (17.8)	Not specified	Not specified	Yes	Age, sex
**Multiple Sclerosis (MS) (*n*** **= 2)**								
Peebles, et al. (2016) [12]	MS	40	28:12	No gait impairment: 45.8 (8.6) Gait impairment: 45.9 (8.7)	Not specified	Not specified	Yes	Not specified
Peebles, et al. (2017) [39]	MS	55	39:16	Non-fallers: 45.9 (9.5) Fallers: 46.6 (10.1)	Not specified	Not specified	Yes	Not specified
**Parkinson’s Disease (PD) (n = 3)**								
Stegemöller, et al. (2012) [37]	PD	10	Not specified	62.0 (9.3)	87.7 (20.5)	1.72 (11.0)	Yes	Age, sex
Catalá, et al. (2016) [36]	PD	25	Not specified	48.0 (5.0)	77.6 (16.6)	1.72 (0.08)	Yes	Age, anthropometric parameters, sport activity level
Martelli, et al. (2017) [11]	PD	9	2:7	64.3 (7.4)	75.5 (15.7)	1.70 (0.06)	Yes	Age
**Miscellaneous (n = 7)**								
McCrum, et al. (2014) [18]	Unilateral peripheral vestibular disorder (UPVD)	17	10:7	49.0 (9.0)	73.8 (14.1)	1.71 (7.3)	Yes	Age, anthropometric parameters, sport activity level, sex
Hoogkamer, et al. (2015) [44]	Cerebellar lesions Pilocytic Astrocytoma (n = 8) Medulloblastoma (n = 5) Astrocytoma grade II (n = 2) Astrocytoma grade III (n = 1) Lhermitte Duclos Disease (n = 1) Hemangioblastoma (n = 1))	18	13:5	24.4 (7.3)	Not specified	Not specified	Yes	Not specified
Rijken, et al. (2015) [40]	Facioscapulohumeral muscular dystrophy	10	3:7	49.0 (5.0)	76.0 (12.0)	1.78 (0.07)	Yes	Age, sex
Ghomian, et al. (2017) [43]	Diabetes Mellitus	1	1	50	Not specified	Not specified	No	n/a
Simon, et al. (2017) [42]	Spinal deformity Lytic spondylolisthesis (*n* = 6) Scoliosis (*n* = 4) Kyphotic deformity (n = 4) Flatback secondary to spinal fusion (n = 2) Degenerative kypho-scoliosis (n = 1)	17	15:2	37.1 (26.0)	Not specified	1.61 (0.02)	Yes	Not specified
van Vugt, et al. (2019) [41]	Hereditary spastic paraparesis	10	4:6	53.5 (11.5)	81.4 (15.2)	Not specified	Yes	Age
de Jong, et al. (2020) [45]	“Balance problems” SCI (n = 15) Stroke (*n* = 15) Other (*n* = 15)	56	18:38	SCI: 57.7 (11.5) Stroke: 54.9 (15.6) Other: 58.8 (14.6)	SCI: 84.1 (6.7) Stroke: 75.3 (19.6) Other: 82.8 (14.3)	SCI: 1.79 (7.3) Stroke: 1.71 (10.3) Other: 1.75 (10.3)	Yes	Not specified

^14^Stroke > 6 months ago

**Table 3:** Gait analysis equipment and conditions

Paper	Gait Analysis Equipment	Marker model	Number of markers	Baseline walking tasks	Gait speed (m/s) [Mean (SD)]	Was treadmill velocity accounted for?
**Stroke (n = 8)**						
Hak, et al. (2013) [14]	CAREN (Motek Medical BV, Amsterdam, the Netherlands) 12 motion capture cameras (120 Hz; Vicon, Oxford, UK)	Lower body Plug-in-Gait	16	4-min treadmill walk	Not specified	Not specified
Kao, et al. (2014) [27]	Instrumented treadmill (1200 Hz; Bertec Corp., Colombus, OH, USA) 8 motion capture cameras (120 Hz; Motion Analysis Corporation, Santa Rosa, CA, USA)	Full-body (Not specified)	46	Three 1-min treadmill walks at 60, 80 and 100% of preferred walking speed and fastest attainable speed.	1.0 (0.3)	Not specified
Hak, et al. (2015) [23]	CAREN (Motek Medical BV, Amsterdam, the Netherlands) 12 motion capture cameras (Vicon, Oxford, UK)	Lower body Plug-in-Gait	16	Six 2-min walks at different percentages of self-selected comfortable walking pace instructing them to adapt my increasing either stride frequency of stride length	Not specified	Not specified
van Meulen, et al. (2016) [20]	Xsens ForceShoes™ (Xsens Technologies B.V., Enschede, The Netherlands) customised with ultrasound sensors	n/a	n/a	Two Timed Up & Go walks at self-selected speeds	0.78 (0.25)	n/a
van Meulen, et al. (2016) [19]	Xsens ForceShoes™ (Xsens Technologies B.V., Enschede, The Netherlands) customised with ultrasound sensors	n/a	n/a	Two 10 m walks at a self-selected speed	0.78 (0.22)	n/a
Vistamehr, et al. (2016) [26]	Instrumented treadmill (1200 Hz; Techmachine, Andrezieux Boutheon, France) 12 motion capture cameras (100 Hz; Vicon, Los Angeles, USA)	Not specified	Not specified	Multiple 30-s treadmill walks at self-selected speed	0.74 (0.27)	Not specified
Punt, et al. (2017) [24]	Gait Real-time Analysis Interactive Lab (GRAIL) (Motekforce Link B.V., NL) 10 motion capture cameras (Vicon, Oxford, UK)	Human Body	47	60 consecutive strides of treadmill walking at 0.41 m/s	0.41	Not specified
Tisserand, et al. (2018) [25]	10 m walkway 12 motion capture cameras (100 Hz; Oqus 7+, Qualisys Sweden)	Plug-in Gait model	35	Treadmill walking at a self-selected speed	0.93 (0.43)	Not specified
**Unilateral transtibial amputation (*n*** **= 5)**						
Curtze, et al. (2011) [33]	Irregular walkway: 8 × 1.5 m custom walkway with triangular prisms under a 3 mm thick carpet. Control walkway: flat laboratory walkway 8 motion capture cameras (100 Hz; Vicon, Oxford, UK)	Full-body Plug-In-Gait	35	4 walks on a flat walkway at a self-selected speed	1.17 (0.13)	n/a
Gates, et al. (2013) [32]	Flat laboratory walkway and 4.2 m × 1.2 m rock surface. Unspecified number of motion capture cameras (120 Hz; Motion Analysis, CA, USA)	6DOF	55	4 walks at 4 different speeds scaled leg length	Not specified	n/a
Hak, et al. (2013) [31]	Computer Assisted Rehabilitation Environment (CAREN, Motek Medical BV, Amsterdam, the Netherlands) 12 motion capture cameras (120 Hz; Vicon, Oxford, UK)	Lower body Plug-in-Gait	16	4-min treadmill walk at self-selected pace	Not specified	Not specified
Beltran, et al. (2014) [30]	CAREN (Motek Medical BV, Amsterdam, the Netherlands) 24 motion capture cameras (60 Hz; Vicon, Oxford, UK)	6DOF	57	5-min treadmill walk at speed relative to leg length	Not specified	Not specified
Hak, et al. (2014) [28]	CAREN (Motek Medical BV, Amsterdam, the Netherlands) 12 motion capture cameras (Vicon, Oxford, UK)	Lower body Plug-in-Gait	16	3.5-min treadmill walk and self-selected speed	1.22 (0.22)	Not specified
**Other amputation (*n*** **= 4)**						
Hof, et al. (2007) [34]	Instrumented treadmill (Entred, Bonte, Zwolle, The Netherlands)	n/a	n/a	2-min walks at each of 3 speeds Normalised to 0.75 m/s, 1 m/s, 1.25 m/s for a leg length of 1.0 m)	Not specified	Not specified
Major, et al. (2013) [3]	10 m level walkway with 6 embedded force plates (960 Hz; AMTI, MA, USA) Unspecified number of motion capture cameras (120 Hz; Motion Analysis, CA, USA)	Lower body Helen Hayes model + right and left acromion process and right and left lateral humeral epicondyle	21	Three walks at 3 different self-selected speeds	Not specified	n/a
Brandt, et al. (2019) [29]	Instrumented treadmill (1000 Hz; Bertec Corp., Colombus, OH, USA) 12 motion capture cameras (100 Hz; Vicon, Oxford, UK)	Not specified	39	Three 1.5-min treadmill walks at self-selected speed	0.73 (0.12)	Not specified
Major, et al. (2019) [35]	Walkway with embedded force plates (960 Hz; AMTI, Watertown, MA, USA) Unspecified number of motion capture cameras (120 Hz; Motion Analysis Corp., Santa Rosa, CA)	Lower body Helen Hayes model + right and left wrist joint, left and right 5th metatarsal, right and left acromion process and right and left lateral humeral epicondyle	26	Five walking trials at self-selected speeds under 3 conditions; (1) without prosthesis, (2) with their own prosthesis and (3) with a mock prosthesis that could have its length, mass and inertial properties altered.	1.2 (0.01)	Not specified
**Spinal cord injury (SCI) (*n*** **= 2)**						
Day, et al. (2012) [38]	Split-belt instrumented treadmill (Tecmachine Inc., Cedex, France) 12 motion capture cameras (100 Hz; Vicon, Oxford, UK)	Plug-In-Gait	Not specified	30-s walks at self-selected speed	0.23 (0.12)	Not specified
Arora, et al. (2019) [13]	10 m walkway with an embedded slip device and force plates (2000 Hz; Advanced Mechanical Technology, Inc., Watertown, MA). 8 motion capture cameras (100 Hz; Vicon Nexus, Vicon Motion Systems, Centennial, CO)	Not specified	63	3 walks at a self-selected speed	0.68 (0.32)	Not specified
**Multiple Sclerosis (MS) (*n*** **= 2)**						
Peebles, et al. (2016) [12]	25 ft. (7.62 m) walkway 8 motion capture cameras (60 Hz; Motion Analysis, CA, USA)	Not specified	12	1 walk at self-selected “preferred”, “slow” and “fast” speeds	Not specified	n/a
Peebles, et al. (2017) [39]	Treadmill (unspecified) Unspecified number of motion capture cameras (60 Hz; Motion Analysis Inc., CA, USA)	Not specified	12	3-min walk at self-selected comfortable speed	Non-fallers: 0.73 (0.23) Fallers: 0.51 (0.30)	Not specified
**Parkinson’s Disease (PD) (*n*** **= 3)**						
Stegemöller, et al. (2012) [37]	9 m walkway 8 motion capture cameras (120 Hz; Vicon, Los Angeles, USA)	Plug-in-Gait	39	5 walks at a self-selected comfortable speed	Not specified	n/a
Catalá, et al. (2016) [36]	12 m walkway 12 motion capture cameras (120 Hz; Vicon, Oxford, UK)	Not specified	21	Walk at 1.3 m/s	1.3	n/a
Martelli, et al. (2017) [11]	Instrumented treadmill (Bertec Instrumented Treadmill) surround by an “Active Tethered Assistive Pelvic Device” to apply perturbations. 10 motion capture cameras (200 Hz; Vicon Bonita, Oxford, UK)	Not specified	55	5-min walk at a self-selected speed	0.89 (0.12)	Treadmill speed was added to the vCoM_AP_
**Miscellaneous (*n*** **= 7)**						
McCrum, et al. (2014) [18]	Treadmill (pulsar 4.0, h/o/ cosmos, Nussdorf-Traunstein, Germany) 8 motion capture cameras (120 Hz; Vicon, Oxford, UK)	Full-body kinematic	26	3–4 min of treadmill walking at 1.4 m/s	1.4 m/s	Treadmill speed was added to the vCoM_AP_
Hoogkamer, et al. (2015) [44]	6 m walkway and treadmill (1000 Hz; custom built instrumented treadmill, Forcelink, Culemborg, The Netherlands) walking at 1.0 m/s Unspecified number of motion capture cameras (100 Hz; Vicon Nexus, Oxford Metrics, Oxford, UK)	n/a	1 cluster at the pelvis	3 walking trials and 3-min treadmill walk	Walkway: 1.12 (0.12); Treadmill: 1.0	Not specified
Rijken, et al. (2015) [40]	10 m walkway 6 motion capture cameras (100 Hz; Vicon, Oxford, UK)	Full body Plug-in-Gait	Not specified	3 walks at a self-selected speed	Mildly affected: 1.24; Moderately affected: 0.82	n/a
Ghomian, et al. (2017) [43]	10 m walkway 6 motion capture cameras (100 Hz; Qualisys Track Manager, Sweden)	Plug-in Gait model	44	1 walking trial for each shoe condition at a self-selected speed	Not specified	n/a
Simon, et al. (2017) [42]	8 m walkway 10 motion capture cameras (120 Hz; Motion Analysis Inc., CA, USA)	Not specified	43	3 walks at a self-selected speed	1.1 (0.1)	n/a
van Vugt, et al. (2019) [41]	10 m walkway 8 motion capture cameras (100 Hz; Vicon, Oxford, UK)	Cleveland Clinic	35	Minimum of 3 walks at self-selected speed	0.95 (0.28)	n/a
de Jong, et al. (2020) [45]	GRAIL (Motekforce Link B.V., NL) 8 motion capture cameras (100 Hz; Vicon, Oxford, UK)	Not specified	12	2-min walk at a self-selected speed	SCI: 0.93 (0.33) Stroke: 0.73 (0.29) Other: 0.91 (0.29)	Not specified

**Table 4:** Summary of XcoM and MoS definitions and calculations. For case-control studies that showed a statistically significant difference and where data was available, Glass’s Δ is reported as a measure of effect size

Paper	CoM definition	Pendulum length	BoS definition	MoS calculation	MoS reference edge	Point of gait	Results as reported in original paper	Standardised results interpretation
**Stroke (n = 8)**								
Hak, et al. (2013) [14]	Centre of the polygon described by 4 pelvic markers	Maximal height of the origin of the pelvis	AP: Lateral malleolar marker of the leading foot	BoS – XcoM	AP: Posterior	AP: Heel strike	No significant group effects.	No significant difference for MoS_ML_ or MoS_AP_.
			ML: Lateral malleolar marker of the leading foot		ML: Lateral	ML: Minimum value per step		
Kao, et al. (2014) [27]	Not specified.	Height of the COM during quiet standing	AP: Front toe marker of the leading foot	BoS - XcoM	AP: Anterior	AP: Heel strike	Post-stroke individuals had smaller average MOS_AP_ (*p* = 0.042) but no difference in MOS_ML_, compared to controls. Post-stroke individuals had greater variability of MOS_AP_ and MOS_ML_ compared to controls (p < 0.001).	Post-stroke individuals had significantly **less stable** MoS_AP_ and no difference for MoS_ML_. Post-stroke individuals had **greater** MoS_AP_ and MoS_ML_ variability, compared to controls. MoS_ML_ variability was significantly **greater** for the affected leg in post-stroke individuals.
			ML: Lateral toe marker of the leading foot		ML: Lateral	ML: Heel strike		
Hak, et al. (2015) [23]	Centre of the polygon described by 4 pelvic markers	Maximal height of the estimated CoM	AP: Heel marker of the leading foot	XcoM - BoS	AP: Posterior	AP: Minimum value per step	MoS_AP_ increased when stride length (p < 0.001) and stride frequency (*p* < 0.001) were increased. MoS_ML_ increased when stride frequency was increased (p < 0.001).	MoS_AP_ increased with increased stride length and stride frequency and MoS_ML_ increased with stride frequency. Increased MoS_AP_ and MoS_ML_ was limited during faster than comfortable stride frequency suggesting inability of post-stroke individuals to regulate MoS using stride frequency.
			ML: Lateral malleolar marker of the leading foot		ML: Lateral	ML: Minimum value per step		
van Meulen, et al. (2016) [20]	Fusion of low-pass filtered CoP data with high-pass filtered double-integrated CoM acceleration data.	Vertical CoM position	AP: Midpoint between the front of each foot	MoS_AP_ = XcoM – BoS MoS_ML_ = |BoS – XCoM|	AP: Anterior	AP: Continuous during double-limb support	A positive, significant correlation was found between fall risk and percentage of time spent with a positive MoS_AP_ (r = 0.75, *p* = 0.014). MoS_ML_ asymmetry was not correlated with participant’s fall risk.	MoS_AP_ was more often **more stable** for more affected post-stroke participants. MoS_AP_ and MoS_ML_ were **less stable** on participants’ affected side.
			ML: Lateral shoe position		ML: Lateral	ML: Continuous during double-limb support		
van Meulen, et al. (2016) [19]	Fusion of low-pass filtered CoP data with high-pass filtered double-integrated CoM acceleration data.	Greater trochanter height estimated from total body height	AP: Line connecting the front of each foot.	XcoM-BoS	AP: Anterior	AP: Continuous during double-limb support	Participants with lower BBS scores tend to have a slower walking speed and small positive average MoS_AP_. There is no significant correlation between BBS and MoS_AP_ (r = 0.41, *p* = 0.167).	MoS_AP_ was not significantly correlated with a standard clinical parameter, but MoS_AP_ was more often **stable** for more affected post-stroke participants.
			ML: n/a		ML: n/a	ML: n/a		
Vistamehr, et al. (2016) [26]	Cumulative anthropometric segmental mass properties (13 segment)	1.34 x leg length (m)	AP: n/a	BoS - XcoM	AP: n/a	AP: n/a	MoS_ML_ was inversely correlated with the clinical scores (BBS and DGI).	MoS_ML_ was significantly moderately negatively correlated with other balance measures **(more stable for lower Berg Balance Score)**. When feet were separated, only the affected side correlated with other balance measures.
			ML: CoP		ML: Lateral	ML: Heel strike		
Punt, et al. (2017) [24]	Cumulative anthropometric segmental mass properties (14 segment)	Not specified	AP: Not specified	BoS - XcoM	AP: Anterior	AP: Heel strike	MoS_AP_ and MoS_ML_ were similar during steady-state gait at a fixed speed for faller and non-faller groups.	MoS_AP_ and MoS_ML_ was not significantly different between faller and non-faller groups for the paretic and non-paretic legs. MoS_AP_ variability was significantly different between faller and non-faller groups for the paretic leg, and for MoS_ML_ variability for the paretic and non-paretic leg.
			ML: Not specified		ML: Lateral	ML: Heel strike		
Tisserand, et al. (2018) [25]	Cumulative anthropometric segmental mass properties (number of segments not specified)	1.34 x leg length (m)	AP: n/a	BoS - XcoM	AP: n/a	AP: n/a	Post-stroke participants had a larger MoS_ML_ than controls during baseline treadmill walking (*p* < 0.01), with a larger MoS_ML_ on the non-paretic side than on the paretic side at ipsilateral foot-strike (*p* < 0.05).	MoS_ML_ was significantly **more stable** for non-paretic and paretic limbs at heel strike compared to controls. MoS_ML_ was significantly **more stable** for the non-paretic limb compared to the paretic limb at heel strike.
			ML: Midpoint between the heel marker and 2nd metatarsal marker		ML: Lateral	ML: Heel strike & toe off		
**Unilateral transtibial amputees (n = 5)**								
Curtze, et al. (2011) [33]	Not specified	1.34 x leg length (m)	AP: n/a	BoS - XcoM	AP: n/a	AP: n/a	There was no significant difference between amputee and control groups for MoS_ML_ (*p* = .763).	MoS_ML_ was not statistically different between amputees and controls, or between prosthetic and sound limbs for the amputee group.
			ML: AP axis defined by the 2nd metatarsal and calcaneal markers		ML: Lateral	ML: Minimum value during stance phase		
Gates, et al. (2013) [32]	Cumulative anthropometric segmental mass properties (number of segments not specified)	1.34 x leg length (m)	AP: n/a	BoS – XcoM	AP: n/a	AP: n/a	Amputees had a greater mean MoS_ML_ than controls (*p* = 0.018). Amputees had a smaller MoS_ML_ on their prosthetic limb compared to intact limb (*p* = 0.036), while controls had no significant between-limb differences.	MoS_ML_ was significantly **more stable** for amputees than controls. Amputees had a significantly **less stable** MoS_ML_ on their prosthetic limb compared to their sound limb.
			ML: 5th metatarsal marker		ML: Lateral	ML: Minimum value during stance phase		
Hak, et al. (2013) [31]	Centre of the polygon described by 4 pelvic markers	Maximal height of the origin of the pelvis	AP: Lateral malleolar marker of the leading foot	BoS - XcoM	AP: Posterior	AP: Continuous	MoS_AP_ was smaller for amputees than for controls (*p* = 0.02). In Amputees had a larger MoS_ML_ than controls (*p* = .013).	MoS_AP_ was significantly **less stable** for amputees than controls. MoS_ML_ was significantly **more stable** for amputees than controls, possibly due to a compensatory wider step width.
			ML: Lateral malleolar marker of the leading foot		ML: Lateral	ML: Continuous		
Beltran, et al. (2014) [30]	Cumulative anthropometric segmental mass properties (13 segment model)	1.34 x leg length (m)	AP: n/a	XcoM – BoS	AP: n/a	AP: n/a	There was no significant difference between mean MoS_ML_ and MoS_ML_ variability between amputees and controls or between intact and prosthetic limbs for the amputee group.	MoS_ML_ was not significantly different between amputees and controls. MoS_ML_ variability was not significantly different between amputees and controls.
			ML: 5th metatarsal marker		ML: Lateral	ML: Minimum value during stance phase		
Hak, et al. (2014) [28]	Centre of the polygon described by 4 pelvic markers	Maximal height of the estimated CoM	AP: Lateral malleolar marker of the leading foot	XcoM - BoS	AP: Posterior	AP: Heel strike & toe off	The MoS_AP_ was significantly larger (p = 0.018) for the sound limb compared to the prosthetic limb. There was a significant (*p* = 0.001) increase of MoS_AP_ between initial contact and contralateral toe-off.	MoS_AP_ was significantly **more stable** at heel strike for the prosthetic limb, compared to the sound limb of amputees, but not significantly different at toe off.
			ML: n/a		ML: n/a	ML: n/a		
**Other amputees (n = 4)**								
Hof, et al. (2007) [34]	Low-pass filter of CoP data	1.34 x trochanter height (m)	AP: n/a	BoS - XCoM	AP: n/a	AP: n/a	In amputees MoS_ML_ for the prosthetic leg was always larger than for the non-prosthetic leg and larger than the values for the control subjects.	MoS_ML_ was significantly **more stable** for amputees compared to controls at all speeds (Glass’s Δ: control vs. prosthetic limb = 1.6; control vs. non-prosthetic limb = 0.3). MoS_ML_ was significantly **more stable** for amputee’s prosthetic limb compared to their sound limb at all speeds.
			ML: CoP		ML: Lateral	ML: Heel strike		
Major, et al. (2013) [3]	Cumulative anthropometric segmental mass properties (number of segments not specified)	Not specified	AP: n/a	BoS - XcoM	AP: n/a	AP: n/a	Amputee step widths were greater than controls at all speeds and prosthetic type (*p* = 0.002). The XcoM exceeded the lateral borders of the BoS in all amputees at fast walk and when using the prosthetic with greater ankle joint motion, but this never happened in controls.	XcoM frequently exceeded the BoS (became **unstable**) in the ML direction for the prosthetic group wearing a prosthetic limb with additional ankle motion compared to controls and the same participants wearing a prosthetic limb with more limited ankle motion where the XcoM was always maintained within the BoS (remained **stable**).
			ML: CoP of the stance limb		ML: Lateral	ML: Peak XcoM		
Brandt, et al. (2019) [29]	Cumulative anthropometric segmental mass properties (number of segments not specified)	1.34 x leg length (m) which was the average of the 2 trochanters	AP: n/a	BoS – XcoM	AP: n/a	AP: n/a	Mean MoS_ML_ was 5.71 cm (1.18 cm) for the prosthetic limb and 4.92 cm (1.18 cm) for the sound limb during baseline treadmill walking.	MoS_ML_ stability was **more stable** for the prosthetic side compared to the intact side, but this was not compared statistically.
			ML: CoP		ML: Lateral	ML: Minimum value per step		
Major, et al. (2019) [35]	Cumulative anthropometric segmental mass properties (12 segment)	1.34 x trochanter height (m)	AP: n/a	BoS – XcoM	AP: n/a	AP: n/a	MOS_ML_ was significantly greater on the sound limb side compared to the prosthetic limb side (*p* = 0.005).	MoS_ML_ was significantly **less stable** for the prosthetic limb compared to the sound limb in all conditions.
			ML: Fifth metatarsal of the stance limb		ML: Lateral	ML: Minimum value per step		
**Spinal cord injury (SCI) (n = 2)**								
Day, et al. (2012) [38]	Cumulative anthropometric segmental mass properties (13 segment model)	Not specified	AP: n/a	BoS - XcoM	AP: n/a	AP: n/a	Participants with SCI had significantly greater MoS_ML_ variability compared to controls (*p* < 0.007).	MoS_ML_ had significantly greater variability in post-SCI participants compared to controls suggesting compensatory control mechanisms to avoid falls.
			ML: CoP		ML: Lateral	ML: Minimum value during double-limb support		
Arora, et al. (2019) [13]	Cumulative anthropometric segmental mass properties (12 segment)	Not specified	AP: Anterior foot boundary	BoS - XcoM	AP: Anterior	AP: Heel strike	MoS_AP_ for participants with spinal cord injury was significantly smaller than controls walking at matched speeds (p < 0.01).	MoS_AP_ was not significantly different between SCI participants and controls walking at their self-selected speed. MoS_AP_ was significantly **less stable** for SCI participants compared to controls walking slower than their self-selected pace to more closely match walking speed on the SCI individuals (Glass’s Δ = 2.9).
			ML: n/a		ML: n/a	ML: n/a		
**Multiple Sclerosis (MS) (n = 2)**								
Peebles, et al. (2016) [12]	Geometric centre of the triangle formed by 2 anterior superior iliac spine markers and the midpoint between the 2 posterior superior iliac spine markers	Distance between the estimated CoM and the ankle marker	AP: Toe marker	BoS – XcoM	AP: Anterior	AP: Heel strike & mid-stance	MS participants with gait impairments had a higher MoS_AP_ than controls (p < 0.001) and MS participants without gait impairments (*p* < 0.001) at heel strike and mid-stance. At heel strike, MS participants with gait impairments had a higher MoS_ML_ than controls (*p* = 0.010).	MoS_AP_ was significantly **more stable** for MS participants with a gait impairment, compared to those without and compared to controls at heel strike and mid-stance (Glass’s Δ: Heel strike = 1.3; Mid-stance = 1.2). MoS_ML_ was significantly **more stable** for the MS participants with a gait impairment compared to controls at heel strike (Glass’s Δ = 1).
			ML: Lateral metatarsophalangeal joint		ML: Lateral	ML: Heel strike & mid-stance		
Peebles, et al. (2017) [39]	Centre of the polygon described by 4 pelvic markers.	Distance between the estimated CoM and the ankle marker	AP: Toe marker	BoS - XcoM	AP: Anterior	AP: Heel strike	MS fallers had a decreased mean MoS_AP_ (p < 0.001) and an increased MoSAP variability (p < 0.001) compared to both MS non-fallers and controls. MS non-fallers had an increased mean MoS_ML_ (*p* = 0.011) compared to controls only, and MS fallers had an increased MoS_ML_ variability (p < 0.001) compared to both MS non-fallers and controls.	MS fallers had **less stable** MoS_AP_ (Glass’s Δ = 1.5) and **increased** MoS_AP_ variability compared to MS non-fallers and controls. MS non-fallers were **more stable** for MoS_ML_ (Glass’s Δ = 0.6) and had increased MoS_ML_ variability compared to controls. MS fallers had increased MoS_ML_ variability compared to MS non-fallers and controls.
			ML: Lateral metatarsophalangeal joint		ML: Lateral	ML: Heel strike		
**Parkinson’s Disease (PD) (n = 3)**								
Stegemöller, et al. (2012) [37]	Cumulative anthropometric segmental mass properties (15 segment)	Distance between the COM and the centre of the ankle joint in the sagittal plane	AP: Marker positions on the foot	BoS - XcoM	AP: Anterior	AP: Heel strike & toe off	At heel strike and toe from the leading and trailing foot the PD group had a significantly larger MoS_AP_ than controls.	PD participants were **more stable** than controls during baseline walking at heel strike and toe off for the leading (Glass’s Δ: Heel strike = 6.9; Toe off = 2.6) and trailing (Glass’s Δ: Heel strike = 8.5; Toe off = 5.3) foot in the AP direction.
			ML: n/a		ML: n/a	ML: n/a		
Catalá, et al. (2016) [36]	Cumulative anthropometric segmental mass properties (number of segments not specified)	Distance between the estimated CoM and the ankle marker	AP: AP distance between the toes of the anterior foot and heel of the posterior foot	BoS - XcoM	AP: Anterior	AP: Heel strike	MoS_AP_ was significantly lower (p < 0.05) in the PD group compared to controls, reflecting more unstable gait patterns in unperturbed walking at the same walking velocity.	MoS_AP_ was significantly **less stable** for PD participants compared to controls.
			ML n/a		ML: n/a	ML: n/a		
Martelli, et al. (2017) [11]	Cumulative anthropometric segmental mass properties (13 segment)	Instantaneous distance between the body CoM and the ankle joint of the leading leg	AP: Tip of the toe	BoS – XcoM	AP: Anterior	AP: Heel strike	PD participants always walked with a significantly lower MoS_AP_ than controls (*p* = 0.044). No significant difference for MoS_ML_.	MoS_AP_ was significantly **less stable** for PD participants than controls. No significant difference for MoS_ML_.
			ML: Fifth metatarsal marker		ML: Lateral	ML: Heel strike		
**Miscellaneous (n = 7)**								
McCrum, et al. (2014) [18]	Cumulative anthropometric segmental mass properties (12 segment model)	Instantaneous distance between the body CoM and the ankle joint of the leading leg	AP: Toe marker of the leading foot	BoS - XcoM	AP: Anterior	AP: Heel strike	No significant differences in MoS_AP_ between UPVD patients and controls. Both groups had a positive MoS_AP_, which indicates a stable body position.	No significant difference for MoS_AP_ between UPVD participants and controls.
			ML: n/a		ML: n/a	ML: n/a		
Hoogkamer, et al. (2015) [44]	Cluster of markers at pelvis	Not specified	AP: n/a	BoS - XcoM	AP: n/a	AP: n/a	No significant different between cerebellar lesion participants and controls for MoS_ML_ during treadmill walking.	MoS_ML_ was not significantly different between cerebellar lesion participants and controls during treadmill walking.
			ML: Lateral boundary of the feet		ML: Lateral	ML: Contralateral toe off		
Rijken, et al. (2015) [40]	Cumulative anthropometric segmental mass properties (12 segment model)	0.55 x body height (m)	AP: Heel marker	BoS – XcoM	AP: Anterior	AP: Heel strike	No significant differences between groups were found in MoS_AP_ or MoS_ML_ values.	No difference in MoS_AP_ or MoS_ML_ for affected participants between mild and moderate severity groups or compared to controls.
			ML: Ankle marker		ML: Lateral	ML: Minimum value during stance phase		
Ghomian, et al. (2017) [43]	Cumulative anthropometric segmental mass properties (15 segment)	Distance between the COM and the lateral heel marker at heel strike	AP: Toe marker	BoS – XcoM	AP: Anterior	AP: Heel strike	MoS_AP_ was significantly different for barefoot condition compared to all three shoe conditions. The barefoot condition had a positive MoS_AP_ while all shoe conditions were negative. No significant differences for mean MoS_ML_ across all conditions.	MoS_AP_ was significantly different for barefoot compared to all shoe trials. MoS_AP_ was **more stable** for barefoot than all rocker shoes. No significant differences were found between any condition for MoS_ML_.
			ML: Lateral rocker or 5th metatarsal marker		ML: Lateral	ML: Heel strike		
Simon, et al. (2017) [42]	Cumulative anthropometric segmental mass properties (13 segment)	Trochanteric height (calculation not specified)	AP: n/a	BoS - XcoM	AP: n/a	AP: n/a	MoS_ML_ was smaller in the spinal deformity group compared to the control group. 14 spinal deformity participants were unstable and the remaining 3 patients were stable.	MoS_ML_ was **less stable** for spinal deformity participants than controls.
			ML: Lateral aspect of the foot created by the line between the lateral toe and lateral malleolar marker		ML: Lateral	ML: Heel strike		
van Vugt, et al. (2019) [41]	Cumulative anthropometric segmental mass properties (number of segments not specified)	Vertical distance between the CoP and the CoM during static trial	AP: Metatarsal marker of the stance foot	BoS - XCoM	AP: Anterior	AP: Heel strike & mid-stance	HSP participants had a significantly lower MoS_ML_ at heel strike and mid-stance. HSP participants had a significantly less negative MoS_AP_ at mid-stance, but there was no difference for MoS_AP_ at heel strike.	HSP participants were significantly **more stable** than controls for MoS_AP_ at mid-stance (Glass’s Δ = 2.1). HSP participants were significantly **less stable** than controls for MoS_ML_ at heel strike and mid-stance (Glass’s Δ: Heel strike = 1.7; Mid-stance = 1.8) .
			ML: 2 cm lateral to the 2nd metatarsal marker		ML: Lateral	ML: Heel strike & mid-stance		
de Jong, et al. (2020) [45]	Centre of the polygon described by 4 pelvic markers.	Maximum height of the CoM	AP: n/a	BoS – XcoM	AP: n/a	AP: n/a	No significant difference for MoS_ML_ between spinal deformity and control groups.	No significant difference between groups for MoS_ML_.
			ML: CoP		ML: Lateral	ML: Minimum value at the start of single-support phase		

### Results of individual studies

#### Clinical populations

Eight studies included participants recovering from a stroke [14, 19, 20, 23–27]. Nine studies included amputee participants; five with unilateral transtibial amputees [28–32], one with bilateral transtibial amputees [3], two with unilateral transfemoral amputees [33, 34] and one with transradial and transhumeral amputees [35]. Participants with Parkinson’s disease were included in three studies [11, 36, 37]. Participants with spinal cord injury [13, 38] and Multiple Sclerosis [12, 39] were included in two studies each. Participants with unilateral peripheral vestibular disorder [18], facioscapulohumeral muscular dystrophy [40], Hereditary Spastic Paraparesis [41], spinal deformity [42], diabetes mellitus [43] and cerebellar lesions [44] were included in one study each. Finally, one study reported a mixed cohort of participants with “balance problems” [45], including; spinal cord injury (*n* = 15), stroke (n = 15), total knee prosthesis (*n* = 3), amputation (*n* = 2) and one of each; brain tumour, contusion, acquired brain injury, autosomal dominant cerebellar ataxia, neuropathic pain, Guillain-Barré syndrome, encephalomyelitis, brain trauma, hereditary spastic paraplegia, vestibular disorder and pain complaints of the ankle and foot. Twenty-two of these studies [3, 11–14, 18, 25, 27, 30–34, 36–41, 44–46] included a control group.

Brief results concerning MoS in the AP (MoS_AP_) and ML (MoS_ML_) directions during straight line walking for each paper are described in Table 4. Below we consolidate results from papers describing stroke survivors and unilateral transtibial amputees because these pathologies were most common. For case-control studies, where groups were significantly different and the data was available, Glass’s Δ is reported to describe the effect size.

#### Post-stroke studies

Eight papers solely focused on post-stroke individuals, and one additional paper had a subset of post-stroke individuals. Understandably participants were generally older, averaging their 60s. Participants were affected by hemiparesis on the left (*n* = 64) or right (*n* = 48), as reported in seven studies. Participants were a mean of 30.3 months (1–111 months) since their stroke. Of the seven studies where it was discernible, two included acute stroke survivors (< 6 months post-stroke) [14, 23] and all other studies included chronic stroke survivors (≥6 months post-stroke). Four studies reported a Berg Balance Scale score (mean: 50.4), 2 studies reported the Fugl-Meyer score (mean: 25.6) 2 studies reported a Functional Ambulation Category (mean: 5.2) and one reported an inclusion criterion of a Functional Ambulation Score of ≥3.

Four papers compared the MoS of post-stroke participants to controls. These papers reported no significant difference in the MoS_ML_ between post-stroke participants and controls at heel strike [27], toe off [25], minimum value per step [14] and minimum value per stance phase [45]. The paper reporting the toe off result [25] also assessed MoS_ML_ at heel strike and reported a significantly bigger (more stable) MoS_ML_ for the post-stroke participants at heel strike [25]. Two of these papers also reported MoS_AP_; one found no difference between groups [14] and one found MoS_AP_ to be significantly smaller (less stable) in post-stroke participants [27]. One paper reported significantly greater MoS_AP_ and MoS_ML_ variability in post-stroke participants, calculated using the standard deviation [27].

Three papers compared the MoS between the paretic and non-paretic limb of post-stroke participants. Two papers compared MoS_ML_; one reported a significantly smaller MoS_ML_ (less stable) on the paretic limb at heel strike [25], and the other reported increased MoS_ML_ variability at heel strike on the paretic limb [27]. One paper reported a trend for MoS_AP_ to be more often greater and positive (unstable) on the paretic limb during double-limb support [20], though no statistical comparison was made.

The MoS_ML_ was found to be significantly moderately correlated with balance measures [26]. Others reported no significant correlation between MoS_AP_ and Berg Balance Scale scores, though overall instability frequency using MoS_AP_ and MoS_ML_ was significantly correlated with Berg Balance Scale scores [19, 20]. See the comment in the ‘Results; Margin of Stability Definition’ section regarding the slightly different methodology in one of these papers [19].

#### Unilateral Transtibial amputee studies

Five papers included unilateral transtibial amputees. Fifty participants had amputations due to trauma, seven were due to vascular incidents, and one each were for limb deficiency, chronic regional pain syndrome and one was unspecified.

Four of the papers compared the transtibial amputees to controls. Two studies reported no difference in minimum MoS_ML_ per stance phase between amputees and controls [30, 33]. One study found that MoS_ML_ was significantly increased (more stable) for the amputee group [32]. Similarly, Hak, et al. (2013) [31] reported significantly greater (more stable) average MoS_ML_ and significantly smaller (less stable) average MoS_AP_ in the amputee group.

Two studies reported no difference in minimum MoS_ML_ per stance phase between the prosthetic and sound limb [30, 33], but one study found that this was significantly decreased (less stable) for the prosthetic limb compared to the sound limb [32]. Hak, et al. (2014) [28] reported that the MoS_AP_ at heel strike was significantly lower (more stable) on the prosthetic limb compared to the sound limb, and found no difference at toe off.

### Equipment used to calculate margin of stability

As described in Table 3, 29 studies collected data in a gait laboratory equipped with a median of ten motion capture cameras (range: 6–24 cameras). The number of motion capture cameras was unspecified in six studies. Motion capture cameras were used to track the trajectories of a median of 35 infrared markers (range: 12–63 markers), most commonly using full-body or lower-limb Plug-In Gait (Vicon, Oxford, UK) models. The number of infrared markers used were unspecified by three studies [26, 38, 40]. Marker trajectories were used to build anthropometric models of each participant with a median of 13 segments (range: 12–15 segments) specified in 13 studies. Two studies used force plate data only to measure MoS [34, 44]. In 15 studies participants walked on a treadmill and in 13 they walked on a flat laboratory surface equipped with embedded force plates, and in one study participants walked on both a treadmill and a flat laboratory surface.

Two studies [19, 20] used custom instrumented shoes (Xsens ForceShoes™; Xsens Technologies B.V., Enschede, The Netherlands) complete with 3D force and torque sensors, 3D inertial sensors and ultrasound transducers. This allowed estimation of relative position, velocity, orientation, and ground reaction forces for each foot, which were used to calculate the MoS. In both studies participants walked on a flat laboratory surface.

### Centre of mass definition

The position of the CoM was estimated using the cumulative mass and position of each anthropometric segment in 18 studies [3, 11, 13, 18, 24–26, 29, 30, 32, 35–38, 40–43], the geometric centre of a polygon created by four pelvic markers in six studies [14, 23, 28, 31, 39, 45], using a fusion of low-pass filtered CoP data with high-pass filtered double-integrated CoM acceleration data in three studies [19, 20, 34], the geometric centre of a triangle created by the left and right anterior superior iliac spine, the mid-point between the left and right posterior superior iliac spine in one study [12] and the position of a cluster of markers on the pelvis in one study [44]. The methodology for CoM position estimation was unspecified in two studies [27, 33].

### Base of support definition

Twenty-five studies measured MoS_ML_. For this calculation, the BoS_ML_ was defined using a lateral toe [27], 2 cm lateral from the 2nd metatarsal marker [41] or 5th metatarsal marker [11, 12, 30, 32, 35, 39, 43], the lateral malleolar marker [14, 23, 31, 40], the lateral position of the shoe [20] or the lateral aspect of the foot defined by the malleolar and lateral toe markers [42] in 15 studies. The BoS_ML_ was defined as the position of the CoP [3, 26, 29, 34, 38, 44, 45] or an approximation of this using the AP axis defined by the position of a toe and heel marker [33] or the midpoint between the heel and 2nd metatarsal marker [25] of the stance limb in nine studies. The BoS_ML_ was not explicitly defined in one study [24].

Eighteen studies measured MoS_AP_. To calculate this, the BoS_AP_ was defined by the toe marker or anterior boundary of the leading foot in seven studies [11–13, 18, 27, 39, 43], by the malleolar marker of the leading foot in 3 studies [14, 28, 31], by the heel marker in 3 studies [23, 36, 40], by the midpoint along the line between the front of each shoe in 2 studies [19, 20] and by a metatarsal marker in 1 study [41]. The BoS_AP_ was not explicitly defined in 2 studies [24, 37].

### Margin of stability definition

One study [19] defined MoS quite differently to other papers, but its similarity permitted its inclusion. In the paper, van Meulen, et al. (2016) describe a Dynamic Stability Margin, similar to MoS_AP_, but where the anterior border of the BoS is the line between the front of both feet and the Dynamic Stability Margin is the shortest distance between that line and the XcoM. As such, their MoS_AP_ is influenced by foot placement rather than CoM progression. As explained below in the ‘Base of Support Definition’ section of the Discussion, the order of the calculation matters less for MoS_ML_ because MoS_ML_ = (− 1)^n^ * (XCoM – BoS).

MoS_ML_ was measured at its minimum value during a specified gait phase in nine studies: during the full gait cycle for each foot in four studies [14, 23, 29, 35]; during the stance phase for each foot in four studies [30, 32, 33, 40]; and during the double support phase in one study [38]. MoS_ML_ was measured at heel strike in twelve studies [11, 24, 26, 27, 34, 39, 42–44], of which two also measured it at mid-stance [12, 41] and toe off [25]. One study measured MoS_ML_ continuously [31], one study measured it at the maximum XcoM_ML_ per step, which usually occurred just after heel strike [3], 1 measured it continuously during the double limb support phase [19] and 1 study measured MoS_ML_ at the start of the single support phase for each foot [45].

MoS_AP_ was measured at heel strike in 14 studies [11, 13, 14, 18, 24, 27, 36, 39, 40, 43], of which two also measured it at mid-stance [12, 41] and toe off [28, 37]. Two studies measured MoS_AP_ continuously [20, 31], one study measured it at its minimum value during the full gait cycle for each foot [23] and one measured it during double foot stance [19].

## Discussion

### Summary of evidence

#### Post-stroke & Unilateral Transtibial Amputee Results

It was not possible to synthesise results for these two groups, partially because the specific objectives of each paper were different, and the primary objective was not always focused on walking in a straight line over a smooth surface. Mostly, the variability in calculation and reporting made synthesis more challenging and no specific conclusions can be made about the MoS_ML/AP_ in either population as a result. It is unclear whether the variability of results is due to measurement method, subject variability or whether the MoS is appropriate for use in pathological populations. Many papers included no control group and numbers included in studies were universally low (mean: 16.5; SD: 13.1). Ideally papers should report an effect size so that the *p*-value can be more accurately considered, though most don’t. Where papers in this systematic review have reported no significant differences between groups, it is possible that they were not sufficiently powered to show a true difference and, as such, may be misleading.

Pathological participants in both post-stroke and amputee papers tend to contain heterogenous populations with characteristics that will affect their stability, such as acute or chronic status post-stroke or the traumatic or acquired nature of an amputation. Many papers included in this systematic review attempt to analyse the ability of participants to adapt to alternate walking conditions, such as on different surfaces, at speeds, whilst completing simultaneous tasks or in response to perturbations and use the MoS among other gait variables to tease these out. Whilst the answers to these questions are important, particularly in relation to fall risk in many of these populations, it would be helpful to first establish a solid baseline information from large, controlled studies using a repeatable and validated measure.

In general, papers reported that unilateral transtibial amputees were either more mediolaterally stable than controls or showed no difference. It is likely that compensatory strategies are employed to achieve this such as changing step width or speed. One paper found amputees to be less stable in the AP direction. For post-stroke participants, papers concluded that they were either more stable, less stable or showed no difference in the ML direction. In the AP direction, papers concluded they were either less stable or showed no difference compared to controls. For both of these pathologies, participant circumstances were quite mixed, so strong generalised conclusions are not advisable at this stage. A notable trend was seen in the stroke and transtibial amputation results that was mirrored in the results of all included studies. For MoS_ML_, when there was a significant difference between cases and controls, the results usually found that cases were more stable than controls. Additionally, when a significant difference was found between paretic and non-paretic or prosthetic and sound limb for MoS_ML_, this usually found that the affected limb was less stable. There are a couple of exceptions to these trends, but the authors feel this information could help contribute to future hypotheses.

At its best, the MoS provides objective data that can be used to report and compare stability amongst pathologies, at different points of the gait cycle, in multiple dynamic situations. Unfortunately, as shown in this review, key methodologies relating to the definitions and calculations of the centre of mass, base of support, and margin of stability are variable, making interpretation and comparison of results challenging. This review cannot draw any definitive conclusions on the MoS in any specific pathology due to different methodology or result interpretation used within a small number of papers with low levels of evidence. We cannot conclude whether the MoS provides better information for certain pathologies, or if some pathologies are more stable than controls (or vice versa), utilising different compensation mechanisms.

#### Centre of Mass definition

Accurately calculating the CoM is the first and most integral step towards calculating the XcoM and subsequent MoS, and inaccuracies at this stage can result in compounding errors [47]. This is particularly pertinent to clinical studies as patients may have atypical anatomy, such as spinal deformities or prosthetic limbs. More rudimentary CoM methods that usually give a good approximation of CoM in healthy populations could incur more errors in a clinical population.

In this systematic review the majority of studies estimated participant’s CoM using the weighted average of the position and mass of each anthropometric body segment derived from a full-body marker set [48]. This method requires a minimum of three non-colinear markers arranged on a plane for each segment (assuming it is rigid). Segment properties are commonly calculated based on cadaveric studies [49–51]. This is arguably the gold-standard method for estimating CoM, though it does still require assumptions to be made regarding anthropometry, rigidity, marker placement, body ‘wobble’ and processing methods [52]. Of course, the additional complexity will add both signal and noise, and increase experimental and post-processing time, and researchers must weigh up these factors to achieve optimal model complexity.

As more markers are required to track anatomical landmarks for each segment, the seven papers that estimated CoM position using only pelvic markers had smaller, lower-body only marker sets. Studies have compared different estimations of CoM such as fewer segments, use of four markers tracking pelvic position and tracking of single markers and found them to be less accurate than gold standard methods [48, 52–54]. Pavei, et al. (2017) [52] showed the four pelvic marker method to be very inaccurate during walking and they discourage its use. The effect of torso and arm movement incurred during dynamic conditions, contributing more than 50% of body mass [49], is likely to have a major impact on the CoM [3] and models that fail to account for this risk inaccuracy. Indeed, Mahaki, et al. (2019) [55] has shown that the ML CoM position plays a vital role in ML foot placement during walking, indicating an ability to predict ML foot placement using ML CoM at up to 85% accuracy during the swing phase. The authors recommend that, when calculating CoM in a pathologic population, the weighted average of the position and mass of each anthropometric body segment is preferable to the pelvic marker method. This is because it is more likely that body posture and conformation might be abnormal, e.g. kyphosis, amputation/prosthesis use, and so the trunk cannot be assumed to be a passive mass sitting squarely atop the pelvis, rather its position is likely to be mobile and/or asymmetrical and contribute dynamically to the position of the CoM.

Forward dynamic methods for estimating CoM position, typically undertaken with fixed equipment in a gait laboratory, are also considered accurate [52], and were used by Hof (2008) [8]. This method is used by four studies in this systematic review, including the two instrumented shoe studies [19, 20], which achieve it using wearable sensors. Forces and moments measured by a sensor on each foot to calculate the trajectory of the CoP, and combining this with the relative foot positions to calculate the CoM position [56]. When compared to the segmental mass method results were satisfactory, though improvements can be made.

#### Base of Support definition

In normal gait, mediolateral stability is predominantly controlled by altering the CoM position using the stance leg or by adjusting the BoS using foot placement of the contralateral limb during swing phase [4]. In his paper, Hof, et al. (2008) [8] described the BoS_ML_ using the position of the CoP, a method used by seven studies and approximated using positional markers by two studies included in this systematic review. Most papers used a lateral foot marker placed in the vicinity of the 5th metatarsophalangeal joint or the lateral malleolus. A foot marker only serves as a functional BoS that assumes the CoP can be instantaneously relocated, whereas using the CoP provides a true mechanical BoS [57].

**Fig. 3:**
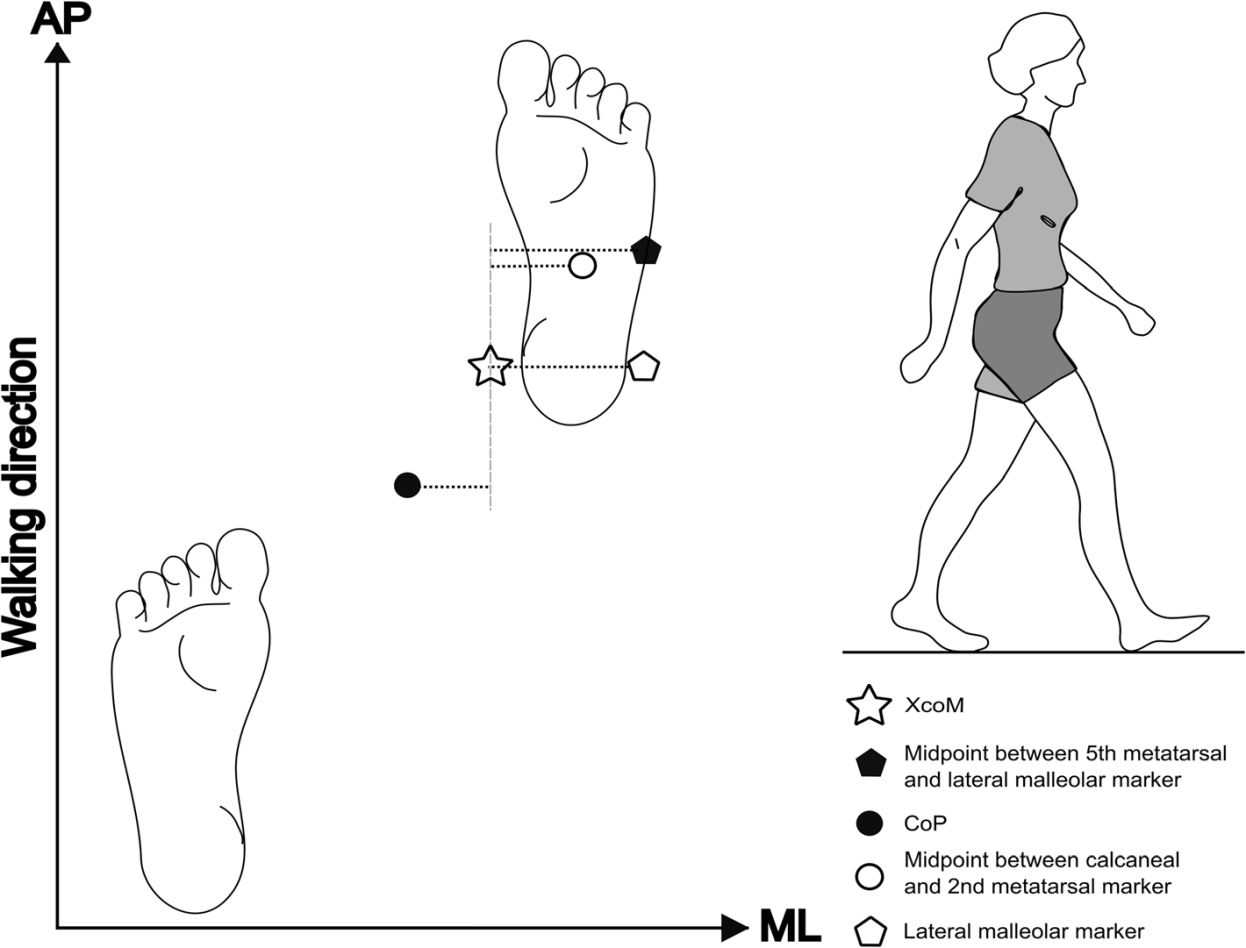
Example of different Base of Support definitions Using heel strike as an example, this diagram shows how different definitions of BoS_ML_ would incur different measurements of MoS_ML_ for the same position of XcoM_ML_ (star). Heel strike has just occurred for the right limb so MoS_ML_ is measured for the right limb. The diagram shows BoS_ML_ measurements taken from the Centre of Pressure [CoP] (black circle), at the midpoint between the 2nd metatarsal and calcaneal marker (red circle), at the lateral malleolar marker (blue circle) and at the midpoint between the 5th metatarsal and lateral malleolar marker (green circle). The MoS_ML_ measurement for each method is shown with a dotted line. This figure was created by FW

Whilst these two methods are similar, the practical application makes a considerable difference. In healthy participants, the position of the CoP snakes anteriorly through the foot from the heel at heel strike to phalange I at toe off, averaging in a central position. During double-limb support the CoP falls somewhere between the feet as pressure is distributed between them. Therefore, when calculating the distance between the XcoM and the BoS_ML_ (MoS_ML_), the difference between, (a) using the position of the CoP or, (b) using the lateral aspect of the foot (via toe or ankle marker) could be more than the diameter of the foot and/or in a different direction, as shown in Fig. 3. Though small, this could be the difference between concluding that the XcoM was “inside” or “outside” the BoS_ML_, a terminology commonly used to describe the participant as stable (XcoM inside the BoS) or unstable (XcoM outside the BoS). Of course, within one study where all measurements are made in the same way and compared to one another this discrepancy matters less, but it makes comparison between studies very challenging. This confusion is further confounded because one foot will generate a positive result, whilst the other generates a negative result. It is very uncommon for any paper to report how they intend to consolidate these results, again meaning that the readers understanding of whether a positive result is stable or unstable difficult and study comparisons challenging.

BoS_AP_ was most commonly measured at the toe marker of the leading foot in an anterior direction, but a few papers were predominantly interested in a ‘backward’ MoS_AP_ measured in the posterior direction from the malleolus or heel as the BoS. In two papers the BoS_AP_ was the midpoint along the line created between the front of the left and right feet. No papers used the position of the CoP to define BoS_AP_. As with BoS_ML_, differences in BoS_AP_ definition makes comparison of results between papers difficult.

#### Margin of Stability definition

Most papers calculate the MoS_AP_ in an anterior direction to consider a forward loss of balance by subtracting the position of the XcoM from the position of the BoS. A handful of studies flip this calculation; usually because they are calculating a ‘backward’ MoS_AP_ in a posterior direction and, as such, a backward loss of balance. In some circumstances a ‘backward’ MoS_AP_ may be more clinically relevant than its opposite. The ‘backward’ MoS method can cause a very slight underestimation of the MoS as the backward boundary is usually the malleolus or heel (where it should be somewhere between the malleolus and heel [7]), which adds another layer of difficulty when trying to compare results. Two papers [19, 20], however, use the ‘backward’ MoS calculation to measure MoS_AP_, but used it with an anterior BoS, which means results are interpreted in the opposite manner, e.g. a positive result would be considered unstable towards a fall in the forward direction, rather than stable, and vice versa. In the mediolateral direction, the calculation is often dependent on the foot; the right foot may be calculated as the BoS – XcoM, while the left foot is calculated as (− 1)*(BoS – XcoM). The (− 1) term corrects for the directionality of the BoS and XcoM vectors and ensures the MoS is positive when the XcoM is medial compared to the BoS.

One paper by de Jong, et al. (2020) [45] describes MoS_ML_ as detailed above, but also describes a “Dynamic Stability Margin” measure, for which the methodology is the same as how two papers [19, 20] described their MoS_AP_ measure. The same paper [45] describes two further measurements called the “XcoM-CoP_AP/ML_”, which are methodologically similar to the MoS_ML_ measurement made by Vistamehr, et al. (2016) [26] and Brandt, et al. (2019) [29]. Due to the variation in BoS and MoS methodology and definition between papers, it is possible that a non-MoS measurement in paper X could bear more likeness to a MoS measurement in paper Y, than a MoS measurement in paper Y does to another MoS measurement in paper Z.

As mentioned in the introduction and throughout the discussion, differences in the definition of the MoS often stem from the direction of the loss of balance, whether left or right for MoS_ML,_ or forward or backward for MoS_AP_. Therefore, we suggest future studies calculate the MoS using the following equation:

$$ MoS=\left(\boldsymbol{BoS}-\boldsymbol{XcoM}\right)\left({\mathbf{e}}_{Instability}\right), $$

where *e*_*Instability*_ is the unit vector in the direction of instability and report the direction of instability for each calculation. Specifying such information would unify the calculation of MoS_AP_ and MoS_ML_, correct for anterior or posterior MoS calculations, and enable methods and interpretations to be clearly communicated.

**Fig. 4:**
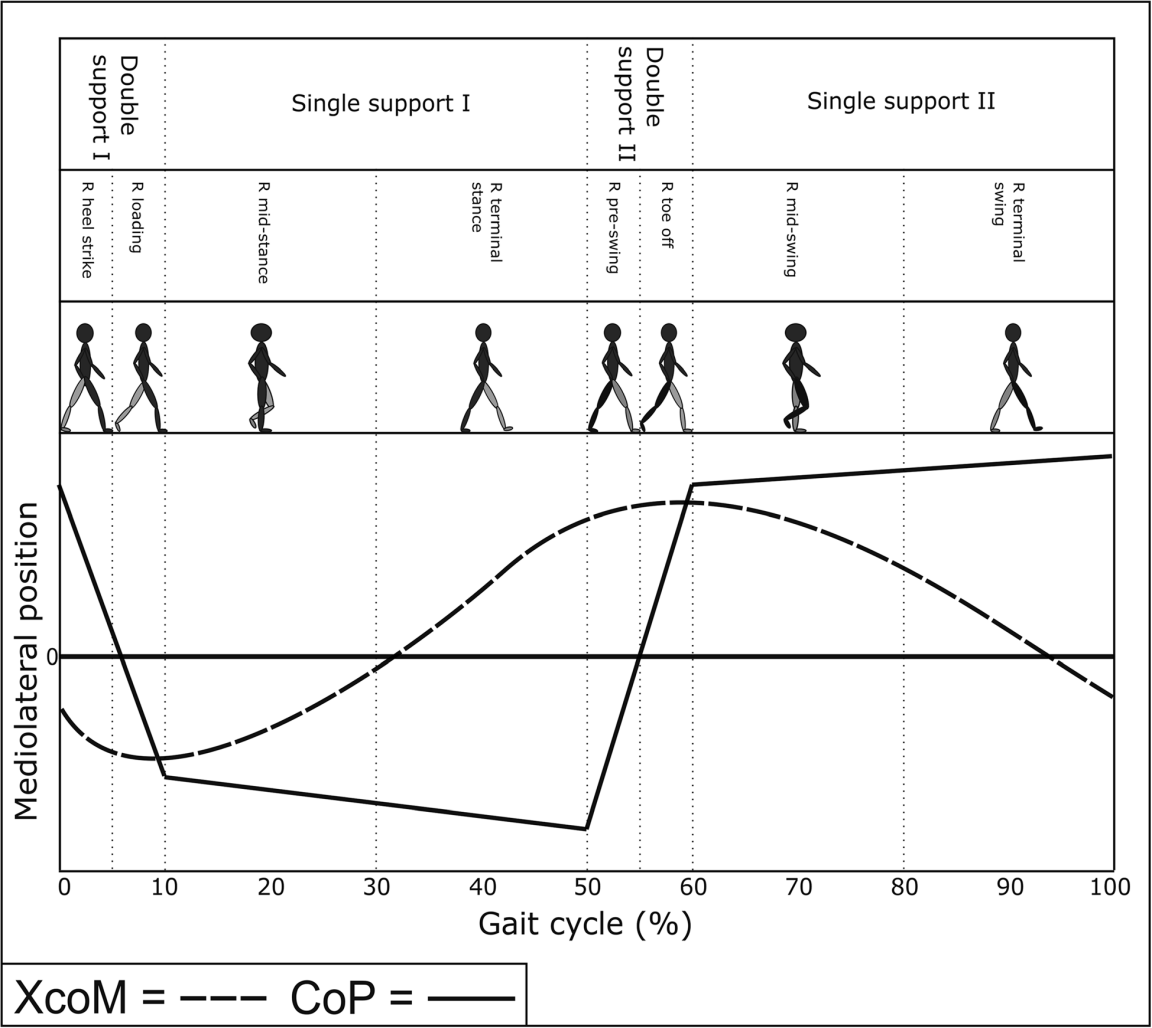
Diagram showing the relationship between CoP and XcoM throughout the gait cycle The CoP (solid line) moves from heel to toe during single support and moves between the feet during double support. The XcoM (dash line) snakes approximately synchronously with the CoP during normal gait. This figure was inspired by Fig. 2 in Day, et al. (2012) [38] and created by FW

The point in the gait cycle at which the MoS_AP/ML_ value is measured varied considerably in the papers reviewed here. The effect of this timepoint on the resulting MoS_ML_ measurement is shown in Fig. 4, based on a figure by Day, et al. (2012) [38]; the MoS would vary greatly depending on the point of the gait cycle at which it was calculated. In Hof’s (2008) [8] paper, MoS_ML_ was calculated at initial foot contact (e.g. heel strike) because, for stable walking, the CoP is placed a certain distance inside or outside of the XcoM so that changes in velocity, turning or stopping can be adapted to. Additionally, Hof’s work was based on instantaneous contact, so the position of the CoP did not change through advancing stance, thus there was no change in BoS. The question remains whether the MoS should be measured at a standardised point of gait, or at the point of gait deemed at most risk of falls for a particular pathologic population being studied.

The velocity at which the MoS was measured should also be considered when interpreting study results, as should the method. If velocity is standardised, participants could be forced to walk at a set speed that is too fast or too slow to be considered comfortable or normal for them, which may affect their stability. Equally however, if participants walk at their own comfortable speed the differences should be accounted for in the analysis and interpretation. In this systematic review, a few treadmill studies scaled velocity to leg length to allow for natural variation in normal speed. Many case-control studies included in this systematic review required participants to walk at a self-selected speed but more than half either did not allow for this in the MoS calculations or statistical analysis or did not report it. Of these, all but one reported a significantly slower velocity for case participants and most calculated MoS_AP_, which is more affected by velocity than MoS_ML_. Potentially, the significant differences (or lack of) reported for MoS_AP_ could be due to gait speed differences rather than stability differences. The most common solution was to account for velocity during statistical analysis, or to match participants by speed (alongside other attributes). Finally, on the topic of velocity, most treadmill studies do not account for belt velocity in their XcoM calculation. Those that do, add the absolute value of the belt velocity to the vCoM within the XcoM calculation reported above in Eq. 1. As with self-selected gait velocity above, this would have the most effect on the MoS_AP_ rather than the MoS_ML_, but it is nonetheless an important omission to consider when comparing studies.

### Limitations

This systematic review only included papers that assessed walking in a straight line as a sole or reported baseline measurement. Straight-line walking was chosen due to its frequency in the literature, likely influenced by the set-up of gait laboratories. Other aspects of walking are important, such as step initiation or termination and turning. Additionally, challenges faced whilst walking in real-life scenario’s such as irregular surfaces and perturbations are also important and worth studying, as are the responses to rehabilitative measures. Furthermore, the study of stability in non-pathologic populations is important to provide normative baseline results across the range of human conditions who still experience a risk of falls, for example, elderly, obese and pregnant people. Finally, a small number of researchers are using the MoS to learn more about children with pathologic gait due to conditions such as cerebral palsy, and further work should consider this population in the context of their developmental stage.

Inherently the MoS is a simplification of human gait and it makes a lot of assumptions due to its foundations in the inverted pendulum model. Foot placement and subsequent stability is the result of complex processing of vision, vestibular and somatosensory inputs, which can be modified by poor mechanical and neural control mechanisms due to neuromuscular pathologies. The inverted pendulum model is a simplification and it’s ‘legs ‘are rigid, so the large effect of joint moments are ignored [14] and it doesn’t allow for possible counter-rotational contributions (e.g. hip torque, upper body motion).

## Conclusions

The MoS has been used to assess stability during straight line walking in many clinical populations, most commonly in amputees and post-stroke individuals, using varying equipment and methodologies. In the papers described here, the MoS has provided good information to the researchers pertaining to the stability and compensatory mechanisms of participants, but numbers are low and populations fairly heterogenous. For clinical application of a measurement, it is important that results can be compared between papers to aid further discovery and benefit patients, which means that measurement and reporting conventions must be established. The biomechanics community should develop standardised reporting guidelines for MoS methodology that recommends inclusion of vital elements such as CoM location and velocity estimation method, pendulum length, gait speed, BoS definition, direction of stability, point of analysis of MoS with respect to the gait cycle and where appropriate; model type, marker set, number of segments, and how treadmill velocity was accounted for. Additionally, efforts to produce a large, controlled baseline of data for distinct patient populations during straight line walking would increase the value of further work on adaptability. The advancement of technology and wearable sensing will no doubt pave the way for more robust datasets in gait laboratories and real-life scenarios.

## Data Availability

Not applicable.

## Abbreviations

AP: Anterior Posterior
BoS: Base of Support
CoP: Centre of Pressure
CoM: Centre of Mass
ML: Mediolateral
MS: Multiple Sclerosis
MoS: Margin of Stability
PD: Idiopathic Parkinson’s Disease
PRISMA: Preferred Reporting Items for Systematic reviews and Meta-Analyses
SCI: Spinal Cord Injury
UPVD: Unilateral Peripheral Vestibular Disorder
XcoM: Extrapolated Centre of Mass
